# *Zonula occludens* 2 and Cell-Cell Contacts Are Required for Normal Nuclear Shape in Epithelia

**DOI:** 10.3390/cells10102568

**Published:** 2021-09-28

**Authors:** Christian Hernández-Guzmán, Helios Gallego-Gutiérrez, Bibiana Chávez-Munguía, Dolores Martín-Tapia, Lorenza González-Mariscal

**Affiliations:** 1 Center for Research and Advanced Studies (Cinvestav), Department of Physiology, Biophysics and Neuroscience, Ave IPN 2508, Mexico City 07360, Mexico; christian485@hotmail.com (C.H.-G.); hgallego@fisio.cinvestav.mx (H.G.-G.); dolores@fisio.cinvestav.mx (D.M.-T.); 2 Center for Research and Advanced Studies (Cinvestav), Department of Infectomics and Molecular Pathogenesis, Ave IPN 2508, Mexico City 07360, Mexico; bchavez@cinvestav.mx

**Keywords:** ZO-2, vimentin, nuclear shape, LINC, SUN-1, senescence

## Abstract

MAGUK protein ZO-2 is present at tight junctions (TJs) and nuclei. In MDCK ZO-2 knockdown (KD) cells, nuclei exhibit an irregular shape with lobules and indentations. This condition correlates with an increase in DNA double strand breaks, however cells are not senescent and instead become resistant to UV-induced senescence. The irregular nuclear shape is also observed in isolated cells and in those without TJs, due to the lack of extracellular calcium. The aberrant nuclear shape of ZO-2 KD cells is not accompanied by a reduced expression of lamins A/C and B and lamin B receptors. Instead, it involves a decrease in constitutive and facultative heterochromatin, and microtubule instability that is restored with docetaxel. ZO-2 KD cells over-express SUN-1 that crosses the inner nuclear membrane and connects the nucleoskeleton of lamin A to nesprins, which traverse the outer nuclear membrane. Nesprins-3 and -4 that indirectly bind on their cytoplasmic face to vimentin and microtubules, respectively, are also over-expressed in ZO-2 KD cells, whereas vimentin is depleted. SUN-1 and lamin B1 co-immunoprecipitate with ZO-2, and SUN-1 associates to ZO-2 in a pull-down assay. Our results suggest that ZO-2 forms a complex with SUN-1 and lamin B1 at the inner nuclear membrane, and that ZO-2 and cell–cell contacts are required for a normal nuclear shape.

## 1. Introduction

*Zonula occludens* 2 (ZO-2) is a protein of the MAGUK (membrane-associated guanylate kinase homolog) family, whose members have PDZ, SH3, and GUK domains (for review, see [1]). The acronym ZO-2 is derived from the *Latin* name for tight junctions (TJs), while the number indicates that it was the second ZO protein identified [2]. ZO-2 is a scaffold protein that, together with ZO-1, is crucial for the polymerization of claudins into TJ strands [3]. ZO-2 functions as a bridge that links the integral TJ proteins—occludin, claudins, and JAM-A—with the cytoskeleton of actomyosin. ZO-2 also works as a platform for the interaction with other peripheral TJ proteins like ZO-1 and cingulin, with proteins of the adherens junction, such as afadin, α- and β-catenins, and with gap junction connexins. In addition, ZO-2 is a hub that concentrates signaling proteins, such as kinases, phosphatases, and GTPases (for review, see [4]).

ZO-2 is a dual localization protein that, in confluent cultures, concentrates at TJs, whereas, in sparse cultures, it is also present at the nucleus [5]. There, ZO-2 is associated with lamin B1, phosphoinositides, chromatin-binding proteins, splicing factors, transcription factors, and transcription regulators (for review, see [4]). ZO-2 functions as a transcriptional repressor of genes involved in cell proliferation, like cyclin D1 [6], and of genes regulated by Wnt signaling [7,8,9], or by Yap, the final effector of the Hippo pathway [10].

Recently, we discovered that the lack of ZO-2 in epithelial MDCK cells triggers an increase in cell size, mediated by the accumulation of Yap at the nucleus, followed by the activation of the PIP3/Akt/mTOR signaling pathway [10]. ZO-2 absence induced the loss of the planar apical network of microtubules, widened paracellular spaces, and the growth of some cells on top of others [11].

Now, we have found in ZO-2 KD MDCK cells a distorted nuclear morphology, characterized by a lobulated appearance. This observation, together with the hypertrophy previously observed in cells lacking ZO-2 [10], led us to investigate if ZO-2 KD MDCK cells display deleterious effects on chromatin and are more susceptible to senescence. The latter is a permanent state of cell cycle arrest that is induced by various factors, including DNA damage, oncogene activation, oxidative stress, chemotherapy, and mitochondrial dysfunction (for review, see [12]).

Our aim has also focused on studying how the lack of ZO-2 triggers a change in nuclear shape. The latter appears to be the result of a balance between chromatin organization and the nucleoskeleton state (for review, see [13]). Lamins A/C and B are type V intermediate filaments that surround the inner nuclear membrane (INM) and form a network that supports the nuclear envelope, allowing the nucleus to resist mechanical perturbations (for review, see [14]). Lamin B1, due to its outer-facing location adjacent to the INM, stabilizes nuclear shape [15]. Likewise, heterochromatin (HC) that is present at the nuclear periphery enhances the ability of the nuclear lamina to maintain the sturdiness and shape of the eukaryotic nucleus [16]. Moreover, the lamin B receptor that spans the INM protrudes into the nucleoplasm and tethers HC to the nuclear periphery, contributing to the maintenance of the shape of interphase nuclei (for review, see [17]). 

Other nuclear envelope proteins maintain nuclear stability by crosslinking the nuclear lamins to the cytoskeleton. This linkage involves the participation of nesprin (nuclear envelope spectrin repeat) proteins located in the outer nuclear membrane (for review, see [18]). Nesprins-1 and -2 connect the nuclear envelope to F-actin; nesprin-3 through plectin binds to intermediate filaments, and nesprin-4 associates with microtubules via kinesin. Nesprins have a KASH domain that mediates their interaction with SUN proteins-1 and -2. These proteins present within the nuclear envelope lumen cross the INM and interact with A-type lamins with their N-terminal domain. Hence, these interactions that span the perinuclear space create a nesprin-SUN complex, known as LINC, that links the nucleoskeleton to the cytoskeleton (for review, see [19]). 

Our results show that although ZO-2 KD cells have an altered nuclear shape and a higher amount of DNA double-strand breaks (DSBs) and are hypertrophic, as previously reported [10], they are not senescent and, instead, are even more resistant to senescence than parental cells. The aberrant nuclear shape is also observed in cells that lack TJs, due to the absence of neighbors or culture in the low extracellular calcium medium, thus indicating that the presence of ZO-2 at both the nuclei and the TJ is required to maintain the nuclear shape. The lack of ZO-2 decreases constitutive and facultative HC but does not reduce the expression of lamins A/C and B, and lamin B receptors. Instead, ZO-2 KD cells over-express SUN-1 and nesprins-3 and -4, silence vimentin and display microtubule instability. ZO-2 is found to associate to lamin B1 and SUN-1, suggesting a new role for ZO-2 in the interaction of the nucleoskeleton with the cytoskeleton.

## 2. Materials and Methods

### 2.1. Cell Culture

Parental (control) and ZO-2 KD MDCK cells were kindly provided by Alan Fanning (University of North Carolina, Chapel Hill, NC, USA) and were cultured as previously described [20] in a DMEM medium (Cat. 31600-083, Life Technologies Corporation, Grand Island, NY, USA) with 10% fetal bovine serum (Cat. S1820-500, Biowest, Riverside, MO, USA), 1% human insulin (Cat. HI0210, Eli Lilly and Company, Indianapolis, IN, USA) and 1% penicillin-streptomycin (Cat. A-01, In Vitro S.A., Mexico City, Mexico) at 37 °C in an atmosphere of 5% carbon dioxide and 95% air. Cells were harvested with 0.05% trypsin-versene solution (Cat. EN-005, In Vitro S.A., Mexico City, Mexico) and plated either sparse (1.5 × 10^5^ cells/cm^2^) or at confluency (3 × 10^5^ cells/cm^2^) in medium with Ca^2+^ (NC; 1.8 mM Ca^2+^). For some experiments, in parental and ZO-2 KD cells were plated at sparse or at confluent density in NC and, after 1 h, were gently washed three times with PBS without Ca^2+^ and incubated in low Ca^2+^ medium (LC; 1–5 μM Ca^2+^) for 20 h. 

The ZO-2 KD cells stably expressed three specific shRNAs against ZO-2 in the pSuper vector, whereas the parental cells expressed the empty vector. Clones of the ZO-2 KD cells were isolated based on their resistance to zeocin. This work was done with the clone IC5, which was previously studied by us [11], and only where specified confirmation experiments were done with clones IC6 and 2D1. In some experiments, ZO-2 was re-expressed in ZO-2 KD cells, using the construct pTRE-hZO-2 resistant to the anti-ZO-2 shRNAs employed (generously provided by Dr. Alan Fanning), as previously described [11]. 

HEK-293T epithelial cells derived from the human embryonic kidney (ATCC, Cat. CRL-3216, Manassas, VA, USA) were grown in a high glucose DMEM medium (Cat. 11965-118, Thermo Fisher Scientific, Waltham, MA, USA) that was supplemented with 5% FBS and penicillin-streptomycin 10,000 (U/μg/mL) (Cat. A-01 In Vitro, Mexico City, Mexico) and were cultured at 37 °C in an atmosphere of 5% carbon dioxide and 95% air.

### 2.2. Quantification of Nuclear Circularity

The CellProfiler software was used to quantify nuclear circularity. Circularity as a form factor was determined using “identify primary objects” followed by “measure object size shape”. Circularity was calculated as 4*π*Area/Perimeter^2^. Value = 1 for a perfectly circular object and decreases as the nuclear shape becomes increasingly convoluted.

### 2.3. Transmission Electron Microscopy

Parental and ZO-2 KD MDCK monolayers were fixed with 2.5% (*v/v*) glutaraldehyde in 0.1 M sodium cacodylate buffer, with a pH of 7.2, for 60 min at room temperature. They were then postfixed for 60 min with 1% (*w/v*) osmium tetroxide in the same buffer. After dehydration with increasing ethanol and propylene oxide concentrations, samples were embedded in Polybed epoxy resins and polymerized at 60 °C for 24 h. Thin sections (60 nm) were contrasted with uranyl acetate and lead citrate, and the observations were done in a JEOL JEM 1011 (Peabody, MA, USA) transmission electron microscope.

### 2.4. DNA Transfection

MDCK ZO-2 KD cells that had reached a 50–60% confluence were transfected with a pTRE-hZO-2 construct using Lipofectamine 2000^TM^ (Cat. 11668-019, Life Technologies, Eugene, OR, USA) according to the manufacturer’s instructions. The complexes were made in DMEM without serum and with a ratio of 1.75:1 μL of Lipofectamine 2000 to μg of plasmid. After 6 h at 37 °C in a CO_2_ incubator, the medium was replaced with complete DMEM and the cells were incubated for an additional 24 h.

To test the transfection efficiency, the ZO-2 KD MDCK cells were transfected with EGFP using the following transfection reagents and the respective manufacturer’s instructions: Lipofectamine 2000^TM^ (Cat. 11668-019, Life Technologies, Eugene, OR, USA); Lipofectamine 3000^TM^ (Cat. L3000-015, Life Technologies, Eugene, OR); TransIT-X2 dynamic delivery system (Cat. MIR 6004, Mirus Bio LLC, Madison, WI, USA); and, TransIT-2020 transfection reagent (Cat. MIR 5404, Mirus Bio LLC, Madison, WI, USA). In addition, we tested electroporation using the electroporator Nucleofector^TM^ II (Lonza Cologne GmbH, Cologne, Germany) and a protocol previously described for MDCK cells [21] that uses an iso-osmolar electroporation buffer (IEB) (KCl 25 mM, KH_2_PO_4_ 0.3 mM, K_2_HPO_4_ 0.85 mM, myo-inositol 280 mOsmol/kg, pH 7.4).

### 2.5. Immunofluorescence

Immunofluorescence was done following standard procedures, as described previously [22]. Briefly, monolayers grown on glass coverslips were fixed with 2% (*w/v*) paraformaldehyde in PBS, pH 7.4, and permeabilized for 15 min with 0.2% (*v/v*) Triton X-100 in PBS. The cells were washed three times with PBS and then blocked for 1 h with 1% (*w/v*) BSA Ig free (Cat. 1331-A, Research Organics, Cleveland, OH, USA). We then used the following primary antibodies: rabbit polyclonal against lamin B1 (Cat. Ab16048, dilution 1:400, Abcam, Cambridge, UK), ZO-2 (Cat. 711400, dilution 1:200, Invitrogen, Carlsbad, CA, USA), H3K9me2 (Cat. 07-441, dilution 1:100, Merck Millipore, Temecula, CA, USA), H3K9me3 (Cat. GTX121677, dilution 1:100, GeneTex Inc., Irvine, CA, USA) and ZO-1 (Cat. 61-7300, dilution 1:300, Invitrogen, Camarillo, CA, USA); or the mouse monoclonals against lamin A/C (Cat. 4777, dilution 1:300, Cell Signaling Technology, Danvers, MA, USA), tubulin (Cat. T7816, dilution 1:100, Sigma Aldrich, St. Louis, MO, USA), vimentin (Cat. Ma5-11883, dilution 1:300, Thermo Fisher Scientific, Rockford, IL, USA) and ϒ-H2AX (Cat. Sc-517348, dilution 1:50, Santa Cruz Biotechnology, Dallas, TX, USA). After being washed three times with PBS, the coverslips were incubated for 1 h at room temperature, with the following secondary antibodies: from donkey against rabbit IgG coupled to Alexa Fluor 488 (Cat. A-21206, dilution 1:1000, Life Technologies, Eugene, OR, USA) or Alexa Fluor 594 (Cat. A-21207, dilution 1:1000, Life Technologies, Eugene, OR, USA); and from donkey against mouse IgG coupled to Alexa Fluor 488 (Cat. A-21202, dilution 1:1000, Life Technologies, Eugene, OR, USA). The cells were then washed three times with PBS and the monolayers were mounted with the antifade reagent Vectashield (Cat. H-1200; Vector Laboratories, Burlingame, CA, USA). Images were acquired on a Leica TCS SP8 laser confocal microscope with a Leica HC PL APO CS2 63x/1.40 oil objective. Emission was collected from 430–490 nm for DAPI, 500–550 nm for Alexa Fluor 488 and 610–675 nm for Alexa Fluor 594, with a pinhole size of 1 AU, a smart offset of 0.2% and a pixel dwell time of 400 ns.

### 2.6. Nuclear Fluorescence Quantification with ImageJ

Using ImageJ, the multi-color images were split into single channels and the color images in single channels were converted to a gray scale. To select the nucleus that was going to be quantified, we then used the DAPI single channel image in gray scale to create a binary image, and subtracted the background of the cytoplasm. Next, in the options section, we selected in the integrated density of the fluorescence, the option of Intden (the product of area and mean gray value), and the single channel image in gray scale that we wanted to analyze. ImageJ then gave a numerical value to the amount of fluorescence present in each analyzed nuclei.

### 2.7. Western Blots

Western blots were done according to standard procedures, as previously reported [23]. Briefly, MDCK cells were lysed at 4 °C with a RIPA buffer (40 mM Tris-HCl, pH 7.6, 150 mM NaCl, 2 mM EDTA, pH 8.0, 10% [*v/v*] glycerol, 1% [*v/v*] Triton X-100, 0.5% [*w/v*] sodium deoxycholate, 0.2% [*w/v*] SDS, and 1 mM phenylmethylsulfonyl fluoride) containing the protease inhibitor cocktail Complete (11697498001; Roche, Mannheim, Germany). Subsequently, the lysates were sonicated three times for 30 s each in a high-intensity ultrasonic processor (Vibra-Cell; Sonics and Materials, Danbury, CT, USA). The proteins in the cellular extracts were quantified, and the samples were diluted in the sample buffer (Cat. NP0008, Invitrogen, Carlsbad, CA, USA), run in 10% polyacrylamide gels, and transferred to polyvinylidene difluoride membranes (GE Healthcare, Little Chalfont, UK). The following primary antibodies were then employed: rabbit polyclonals against lamin B1 (Cat. Ab16048, dilution 1:5000, Abcam, Cambridge, UK), anti glyceraldehyde-3-phosphate-dehydrogenase (GAPDH) (Cat. RPCA-GAPDH, dilution 1:50,000, Encore Biotechnology Inc. Gainesville, FL, USA), anti nesprin-3 (Cat. STJ94404, dilution 1:1000, ST John’s Laboratory, London, UK), anti nesprin-4 (GTX46600, dilution 1:1000, GeneTex Inc., Irvine, CA, USA), anti-SUN-1 (GTX45959, dilution 1:500, GeneTex Inc., Irvine, CA, USA), H3K9me3 (Cat. GTX121677, dilution 1:1000, GeneTex Inc., Irvine, CA, USA), and anti H3K9me2 (Cat. 07-441, dilution 1:500, Merck Millipore, Temecula, CA, USA); as well as mouse monoclonals against vimentin (Cat. Ma5-11883, dilution 1:1000, Thermo Fisher Scientific, Rockford, IL, USA), lamin A/C (Cat. Sc-398927, dilution 1:500, Santa Cruz Biotechnology, Dallas, TX, USA), lamin B receptor (Cat. Ab32535, dilution 1:1000, Abcam, Cambridge, MA, USA) and ϒ-H2AX (Cat. Sc-517348, dilution 1:1000, Santa Cruz Biotechnology, Dallas, TX, USA); and a rabbit monoclonal anti-p21 (Cat. 2947T, dilution 1:1000, Cell Signaling Technology, Danvers, MA, USA). As secondary antibodies, we employed peroxidase-conjugated goat antibodies against the rabbit IgG (Cat. 62-6120, dilution 1:20,000, Invitrogen, Camarillo, CA, USA) or mouse IgG (Cat. 62-6520, dilution 1:10,000, Invitrogen, Camarillo, CA, USA) and used the chemiluminescence detection system Immobilon™ western (Merck Millipore, Cat. WBKLS 0500, Darmstadt, Germany).

### 2.8. Western Blot Densitometry with ImageJ 

Using ImageJ, the Western blot image was converted to a gray scale. The square selection tool was then employed to highlight the first lane and to set a rectangle in the area to analyze. Next, we selected the rectangle of the first lane and dragged it over to the next lane, set it in place, and repeated this procedure for each subsequent lane on the gel. Finally, we selected the option of plot lanes. Western blots always have some background signal and hence the start and the end of the peaks that quantitate the intensity of the signal in each lane do not reach the baseline of the profile plot. Therefore, to discard the background of each lane, a line was drawn across the base of the peak to close it off, as has been previously described in the following link: https://lukemiller.org/index.php/2010/11/analyzing-gels-and-western-blots-with-image-j/ (accessed on 19 August 2021).

With the wand tool, we then selected inside the peak and the measurements were displayed in the results window. To calculate the relative density of each band, its value was divided by the value of its standard (e.g., GAPDH).

### 2.9. Immunofluorescence Detection of β-Galactosidase Activity 

The activity of β-galactosidase was evaluated through its most common substrate, 5-Bromo-4-chloro-3-indolyl β-d-galactopyranoside (X-gal), which produces a dark blue precipitate that can be detected by light microscopy [24] or by confocal fluorescence based on the fact that the X-gal precipitate absorbs light in the 570–700 nm range and emits fluorescence in the 650–770 nm range [25]. Here, we employed the confocal fluorescence detection method previously described [25], as it allows identification of X-gal-labeled cells. Briefly, parental and ZO-2 KD MDCK cells grown on glass coverslips were fixed in 3% formaldehyde for 5 min at room temperature, permeabilized for 15 min with 0.25% (*v/v*) Triton X-100 in PBS, and then blocked for 1 h with 1% (*w/v*) bovine serum albumin IgG free. The cells were then washed three times with PBS and incubated overnight at 37 °C (non CO_2_) with X-gal staining solution (5 mM K_3_Fe(CN)6, 5 mM K_4_Fe(CN)6, 2 mM MgCl_2_, 50 µg/mL X-gal, in citric acid/Na phosphate buffer pH 6.0). After X-gal staining, the monolayers were incubated overnight at 4 °C with a rabbit antibody against ZO-1 (Cat. 61-7300, dilution 1:300, Invitrogen, Camarillo, CA, USA) to detect the cell borders. As a secondary antibody, we employed, for 1 h at room temperature, a donkey against rabbit IgG coupled to Alexa Fluor 488 (Cat. A-21206, dilution 1:1000, Life Technologies, Eugene, OR, USA). The cells were excited at 633 nm, and the fluorescence emitted by X-gal was recorded at 650–770 nm. A Leica TCS SP8 confocal microscope was employed. 

### 2.10. Immunofluorescence Detection of Apoptosis

ZO-2 KD and parental MDCK cells subjected to the UV radiation assay were tested for apoptosis with the TUNEL assay (Cat. 11 684 795 910, Roche Diagnostics, Mannheim, Germany), according to the manufacturer’s instructions. Staurosporine was employed for 24 h at a concentration of 2 μM as a positive control to induce apoptosis

### 2.11. Pull-Down Assays

HEK293T cells were transfected with amino (398–962 nt), 3PSG (1595–3019 nt), or AP (3029–3923 nt) segments of cZO-2, in the pcDNA4/HisMax vector that had been reported previously [26]. After 24 h, the cells were lysed, and the extracts were subjected to affinity chromatography with Complete His-Tag Purification Columns (Cat. COHISC-RO, Sigma Aldrich, St. Louis, MO, USA), following the manufacturer’s instructions. The purified fractions were run in an SDS-PAGE and blotted with rabbit polyclonal antibodies against 6x His tag (Cat. GTX115045, dilution 1:5000; Genetex, Irvine, CA, USA), anti the amino segment of ZO-2 (Cat. 71-1400, dilution 1:100; Invitrogen, Carlsbad, CA, USA), anti the C-terminal region of ZO-2 (Cat. 00238, dilution 1:1000; BiCell Scientific, Maryland Heights, MO, USA) and SUN-1 (GTX45959, dilution 1:500, GeneTex Inc., Irvine, CA, USA).

### 2.12. Cell Fractionation Assay

Cytoplasmic and nuclear fractions of parental MDCK cells were obtained, as previously reported [27]. Briefly, cells were lysed in an isotonic buffer A (40 mM HEPES, pH 7.4, 120 mM KCl, 2 mM EGTA, 0.4% glycerol, 10 mM β-glycerophosphate and 0.2% NP-40) and were set to rotate for 30 min at 4 °C. Nuclei were pelleted by centrifugation at 1000× *g* for 5 min. The supernatant was centrifuged further at 10,000× *g* for 10 min in order to obtain the cytosolic fraction. The nuclear pellet was then gently washed with 0.1% NP-40 and buffer A without detergent. Samples were then centrifuged at 1000× *g* for 5 min. The supernatant was discarded, and the nuclear pellet was resuspended in the RIPA buffer.

### 2.13. Drugs 

Nocodazole (Cat. M-1404, Merck Millipore, Darmstadt, Germany) was prepared as a 10 mM stock in DMSO and used at a concentration of 10 μM.

Docetaxel (Cat. 01885, Merck Millipore, Darmstadt, Germany) was prepared as a 10 mM stock in DMSO and used at a concentration of 10 μM.

Staurosporine (Cat. S4400, Sigma Aldrich, St. Louis, MO, USA) was prepared as a 100 μM stock in DMSO and used at a concentration of 2 μM.

### 2.14. Statistical Analysis

Data of the integrated density of fluorescence and Western blot densitometry derived from parental, ZO-2 KD, and ZO-2 KD + hZO-2 MDCK cells were compared for statistically significant differences. For normally distributed data and equal variances we applied Student’s *t*-test for the analysis of two groups of results, and one-way ANOVA followed by Tukey’s post-hoc test for multiple comparisons of three or more groups of data. When the results displayed a normal distribution and unequal variances, we applied One-way ANOVA with Welch correction, followed by Games-Howell’s comparisons test. For not normally distributed data, we employed the Mann-Whitney test for the comparison of two sets of data, and the Kruskal-Wallis test with the multiple comparison Dunn’s test for three or more sets of data. Graphs were generated using GraphPad Prism 6.01 software.

## 3. Results

### 3.1. In ZO-2 KD MDCK Cells, the Nuclei Are Lobulated and Display Multiple Indentations

**Figure 1:**
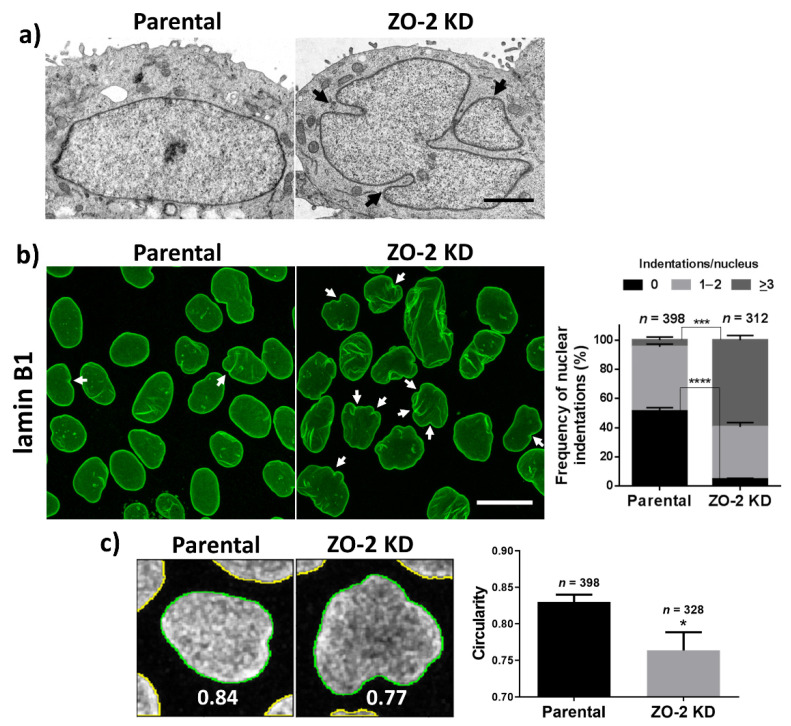
The nuclei of ZO-2 KD MDCK cells are lobulated and have multiple indentations. (**a**) Transmission electron microscopy of parental and ZO-2 KD MDCK cells. Arrows, nuclear lobulations. Bar, 20 μm. (**b**) Immunofluorescence of parental and ZO-2 KD MDCK cells treated with an antibody against lamin B1. Left panel, a representative image of one optical section per experimental condition; arrows, nuclear indentations. Bar, 20 μm. Right panel, quantitative analysis. Statistical analysis was done with Student’s *t*-test. Results shown as media ± standard deviation *** *p* = 0.001; **** *p* < 0.0001. (**c**) Quantification of nuclear circularity. Left panel, a representative image of quantification of nuclear circularity in parental and ZO-2 KD MDCK cells stained with lamin B1. Right panel, results obtained from three independent experiments. *n*, number of nuclei evaluated. Statistical analysis was done with Student’s *t*-test. Results are shown as media ± standard deviation * *p* = 0.0130.

We observed with transmission electron microscopy that, in contrast to parental MDCK cells, the nuclei of ZO-2 KD cells are lobulated (segmented) (Figure 1a). In addition, the ZO-2 KD cells incubated with antibodies against lamin B1, which localizes at the INM, display by immunofluorescence multiple indentations at the nuclear envelope (Figure 1b). Moreover, when we evaluated the degree of nuclear circularity, where values closer to 1 indicate a higher circularity, we detected lower values in ZO-2 KD MDCK cells in comparison to parental cells (Figure 1c). We observed this same pattern of nuclear lobulations and indentations in three different clones of ZO-2 KD cells (Figure S1).

### 3.2. The Lack of ZO-2 Increases the Phosphorylation of Histone H2AX, Indicative of More Abundant DNA Double Strands Breaks

**Figure 2:**
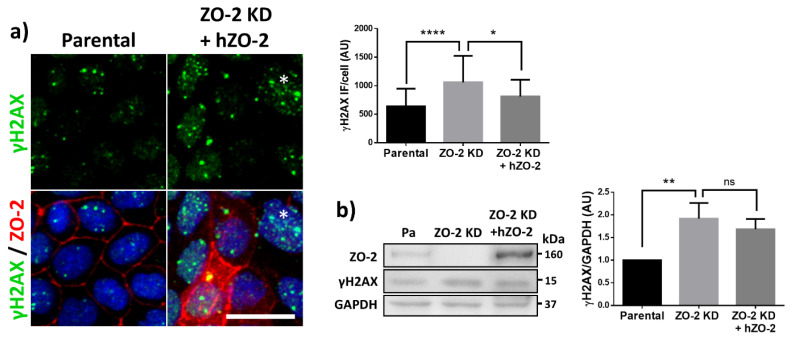
ϒ-H2AX nuclear foci are more abundant in ZO-2 KD than in parental MDCK cells. (**a**) Parental and ZO-2 KD MDCK cells transfected or not with hZO-2 were processed for immunofluorescence with an antibody against ϒ-H2AX, a DDR marker. Cell borders were stained with antibodies against ZO-1, and the nuclei were detected with DAPI. Left, representative images, bar 20 μm; *, non-transfected cell. Right, quantitative analysis done with ImageJ. Data obtained from at least 38 cells derived from three optical fields in each experimental condition: parental cells, MDCK ZO-2 KD cells highly expressing transfected hZO-2, and ZO-2 KD cells without visible transfected hZO-2 signal. Statistical analysis was done with Kruskal-Wallis followed by Dunn’s multiple comparisons test. Results are shown as media ± standard deviation. * *p* < 0.05, **** *p* < 0.0001. (**b**) Western blot detection of ϒ-H2AX in Parental and ZO-2 KD MDCK cells transfected or not with hZO-2. GAPDH was employed as a loading control. Left panel, representative Western blot; right panel, quantitative analysis of three independent experiments. Statistical analysis was done with one-way ANOVA, followed by Tukey’s multiple comparisons test. Results are shown as media ± standard deviation, ** *p* < 0.01; ns, non-significant.

To find if a deleterious effect on chromatin accompanied this change in nuclear shape, we analyzed the phosphorylation of histone H2AX (ϒ-H2AX) in parental and ZO-2 KD MDCK cells transfected or not with ZO-2. H2AX phosphorylates, due to the appearance of DNA DSBs that activate the DNA damage response (DDR). DSBs induce the recruitment and binding of the kinase ataxia-telangiectasia mutated (ATM) to the sites of DNA damage, driving the histone H2AX phosphorylation [28] that favors the assembly of DNA-repair complexes (for review see [29]). Figure 2a shows, by immunofluorescence, that the expression of the nuclear foci of ϒ-H2AX increases in ZO-2 KD MDCK cells not transfected with ZO-2, and diminishes to control values in ZO-2 KD cells transfected with an hZO-2 construct not vulnerable to the shRNAs against ZO-2. By Western blot, we confirmed the increase in ϒ-H2AX expression in ZO-2 KD cells (Figure 2b). However, we could not detect, by Western blot, a recovery after hZO-2 transfection in ZO-2 KD cells. This could be because the accumulation of DSB rises as a long-term effect due to the absence of ZO-2, which may not be repairable in the short term after the reintroduction of ZO-2 in the cells. Alternatively, it could be due to the fact that in the Western blot experiments we quantitate the amount of ϒ-H2AX present in the whole monolayer, and as can be seen by immunofluorescence (Figure 2a), only a small percentage of cells in the monolayers are transfected. To increase transfection efficiency, we tested electroporation and a variety of transfection reagents, but found that none could increase it above the 20% efficiency obtained with the Lipofectamine 2000 reagent employed throughout our experiments (Figure S2).

### 3.3. ZO-2 KD Cells Are More Resistant to Senescence Than Parental Cells

**Figure 3:**
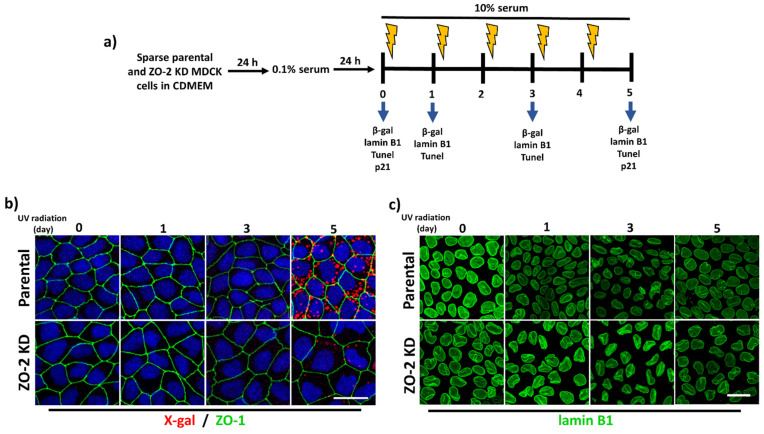

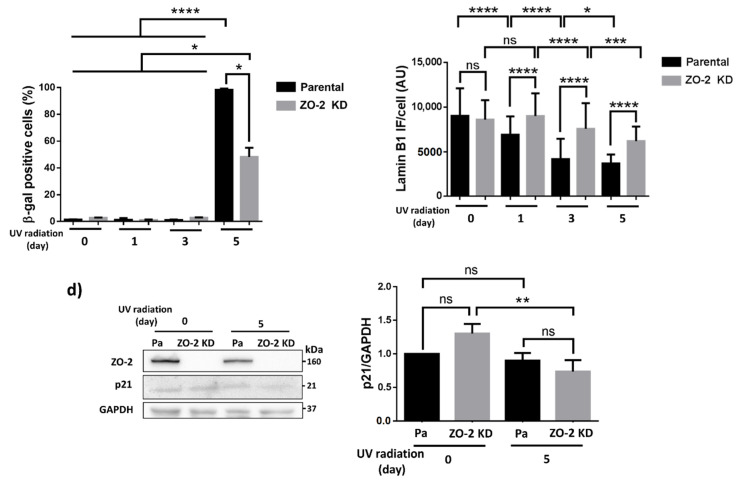
UV radiation-induced senescence is lower in ZO-2 KD cells than in parental cells. (**a**) Schematic representation of the UV-C irradiation treatment applied to parental and ZO-2 KD MDCK cells. Sparse cultures of parental and ZO-2 KD cells were incubated in CDMEM for 24 h and then transferred for one more day in media with 0.1% fetal calf serum. After that, the monolayers were transferred to media with 10% serum and irradiated for 2 sec with UV-C light (2 J/m^2^) every day for 5 consecutive days. Monolayers were retrieved for immunofluorescence detection of β-galactosidase (β-gal) or lamin B1 at days 0, 1, 3, and 5. (**b**) The activity of β-gal is lower in ZO-2 KD cells than in parental cells. The activity of β-gal was evaluated through its substrate X-gal that produces a dark blue precipitate that can be excited at 633 nm and emits fluorescence at 650–770 nm range. Monolayers were also treated with antibodies against ZO-1 to detect the cell borders, and the nuclei were stained with DAPI. Upper panel, representative images, bar, 20 μm; lower panel, quantitative analysis done with ImageJ. Data obtained from three fields in each experimental condition. Statistical analysis was done with One-way ANOVA with Welch correction followed by Games–Howell’s comparisons test. Results are shown as media ± standard deviation. * *p* < 0.05, **** *p* < 0.0001. (**c**) The decrease in lamin B1 expression triggered by UV radiation-induced senescence is delayed in ZO-2 KD cells. Lamin B1 immunofluorescence was detected with a specific antibody. Upper panel, representative images, bar, 20 μm; lower panel, quantitative analysis done with ImageJ. Data obtained from at least 150 cells per condition derived from three fields in each experimental condition. Statistical analysis was done with Kruskal-Wallis followed by Dunn’s multiple comparisons test. Results are shown as media ± standard deviation. ns, non-significant, * *p* < 0.05, *** *p* = 0.001, **** *p* < 0.0001. (**d**) Western blot detection of p21 in parental and ZO-2 KD MDCK cells at 0 and 5 days after UV radiation. GAPDH was employed as a loading control. Left panel, representative Western blot; right panel, quantitative analysis of three independent experiments. Statistical analysis was done with one-way ANOVA followed by Tukey’s multiple comparisons test. Results are shown as media ± standard deviation, ** *p* < 0.01; ns, non-significant.

The altered nuclear shape, the increase in ϒ-H2AX nuclear foci, and the hypertrophy previously observed in ZO-2 KD cells [10] prompted us to explore if the absence of ZO-2 made cells more prone to senescence induced by UV-radiation. For this purpose, we transferred sparse cells cultured in CDMEM for 24 h to serum-depleted media in order to synchronize the culture. After 24 h, the monolayers were incubated in media with 10% serum and irradiated every day for 2 s with UV-C light (2 J/m^2^) during five consecutive days (Figure 3a). To analyze senescence development, we detected the activity of β-galactosidase as a marker of the enhanced lysosomal content of senescent cells [30]. The β-galactosidase is optimally active at pH 4.0–4.5; hence its detection at suboptimal pH 6.0 denotes its very high expression level in senescent cells [30]. Figure 3b shows that the number of red fluorescent spots of β-galactosidase activity detected with the artificial substrate X-gal increased in parental cells as the number of days with UV-light treatment augmented. However, in ZO-2 KD cells, even after five consecutive days of UV-radiation, the red fluorescent spots of β-galactosidase activity were barely detectable (Figure 3b). 

Another hallmark of senescence is the loss of nuclear lamin B1 [31]. Hence, we next analyzed in the UV-radiation assay the expression of lamin B1, finding as expected that in parental MDCK cells, the lamin B1 signal diminished as the number of days with UV-light treatment accumulated. However, in ZO-2 KD MDCK monolayers, the decrease in lamin B1 expression was delayed (Figure 3c). 

In fibroblasts, another marker of senescence coupled to the increase in β-galactosidase activity is the activation of p53/p21 pathway [32]. Therefore, we next tested p21 expression in UV-radiated parental and ZO-2 KD MDCK cells. The Western blot in Figure 3d shows the same level of expression of p21 in parental and ZO-2 KD cells at zero days of radiation. However, in ZO-2 KD cells the amount of p21 diminishes after five days of radiation, while in parental cells no change is observed. These results suggest that ZO-2 KD cells are not senescent and are even more resistant to senescence induced by UV radiation than parental cells.

In addition, we analyzed with a TUNEL (Terminal deoxynucleotidyl transferase dUTP Nick-End Labeling) assay whether ZO-2 KD cells are more sensitive than parental cells to apoptosis. Our results show that neither parental nor ZO-2 KD cells display apoptosis before or after UV radiation, and that this process can only be detected in cells treated for 24 h with staurosporine 2 μM (Figure S3).

### 3.4. In ZO-2 KD MDCK Cells, the Expression of Lamins A/C and B, and Lamin B Receptor Is Unaffected

**Figure 4:**
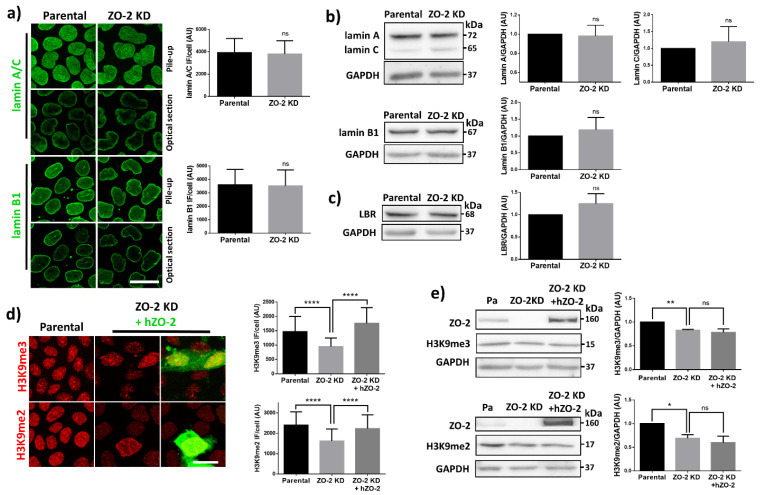
In ZO-2 KD MDCK cells, lamins A/C and B1 and lamin B receptors are unaffected, while the expression of constitutive and facultative heterochromatin diminishes. (**a**) Monolayers of parental and ZO-2 KD MDCK cells were processed for immunofluorescence with antibodies against lamins A/C and B1. Left panels, representative images obtained of pile-ups or single sections; bar, 20 μm. Right, quantitative analysis was done with ImageJ of pile-up images. Data obtained with 200 nuclei in parental condition and 141 nuclei in ZO-2 KD condition for lamin A/C, and 380 nuclei per experimental condition for lamin B1, from two independent experiments. Statistical analysis was done with Mann–Whitney test. Results are shown as media ± standard deviation. ns, non-significant. (**b**) Western blot analysis of lamins A/C and B1 in parental and ZO-2 KD MDCK cells. GAPDH was employed as a loading control. Left, representative images; right quantitative analysis. Results from three independent experiments. Statistical analysis was done with Student’s *t*-test. Results are shown as media ± standard deviation. ns, non-significant. (**c**) Western blot analysis of lamin B receptor (LBR) in parental and ZO-2 KD MDCK cells. GAPDH was employed as a loading control. Left, representative images; right quantitative analysis. Results from three independent experiments. Statistical analysis done with Student’s *t*-test. Results are shown as media ± standard deviation. ns, non-significant. (**d**) Immunofluorescence analysis of constitutive HC marker H3K9me3 and facultative HC marker H3K9me2 in parental and ZO-2 KD MDCK cells transfected or not with hZO-2. Left, representative images; bar, 20 μm. Right, quantitative analysis was done with ImageJ. Data obtained from at least 60 cells per condition: parental cells, MDCK ZO-2 KD cells highly expressing transfected hZO-2, and ZO-2 KD cells without visible transfected hZO-2 signal. Statistical analysis was done with Kruskal-Wallis followed by Dunn’s multiple comparisons test. **** *p* < 0.0001. Results are shown as media ± standard deviation. (**e**) Western blot analysis of H3K9me3 and H3K9me2 in parental and ZO-2 KD MDCK cells transfected or not with hZO-2. GAPDH was employed as a loading control. Representative Western blots are shown together with the quantitative analysis. Results from three independent experiments. Statistical analysis done with One-way ANOVA followed by Tukey’s multiple comparisons test. Results are shown as media ± standard deviation. * *p* < 0.05; ** *p* < 0.01; ns, non-significant.

Next, we studied the mechanism through which the lack of ZO-2 alters nuclear shape. Since changes in nuclear shape relate to an increase in the nuclear envelope’s flexibility [33], we analyzed the level of expression of lamins in ZO-2 KD cells. By immunofluorescence and Western blot, we detected no changes in the level of expression of lamins A/C and B1 between parental and ZO-2 KD MDCK cells (Figure 4a,b). Nuclear lobulation can be accompanied by an increase in lamin B receptors [33]. However, by Western blot, we found no difference in the amount of the lamin B receptor present between the parental and ZO-2 KD cells (Figure 4c).

These results indicate that the change in nuclear shape in the ZO-2 KD cells is not due to a reduced lamin content and does not suggest an altered connection of the lamin B receptors to the underlying HC.

### 3.5. The Lack of ZO-2 Induces a Decrease in Constitutive and Facultative Heterochromatin in MDCK Cells

Two forms of chromatin are present in the interphase nucleus: diffuse euchromatin (EC) that contains transcriptionally competent genes and condensed heterochromatin (HC) where transcription is repressed. HC at the nuclear periphery maintains nuclear shape [16,34] and, accordingly, chromatin histone modification state is a significant determinant of nuclear morphology [35] (for review, see [13]). Hence, we next asked if, in ZO-2 KD cells, the nuclear shape change could be due to a decrease in constitutive HC that generates softer nuclei. For this purpose, we analyzed the expression of the constitutive HC marker H3K9me3 (tri-methylation of lysine 9 on histone 3) on the nuclei of parental and ZO-2 KD cells. Figure 4d (upper panels) shows by immunofluorescence that this constitutive HC marker diminishes in ZO-2 KD cells, compared to parental cells. Transfection of ZO-2 KD cells with an hZO-2 construct not vulnerable to the shRNAs against ZO-2 allowed the recovery of the expression of H3K9me3. By Western blot, we confirmed the decreased expression of H3K9me3 in ZO-2 KD cells (Figure 4e); however, we could not detect a recovery after hZO-2 transfection in ZO-2 KD cells, probably due to the low transfection efficiency that hides the rescue of the phenotype in the Western blot. 

In addition, we analyzed the expression of H3K9me2 (di-methylation of lysine 9 on histone 3), which is a marker of facultative HC that switches between HC and EC states according to the biological context, in contrast to the permanent state of constitutive HC found in centromeres and telomeres [36]. Figure 4d (lower panels) shows by immunofluorescence that the expression of this facultative HC marker also diminishes in ZO-2 KD cells. Transfection of ZO-2 KD cells with the hZO-2 recovered the expression of H3K9me2. By Western blot, we did not see the recovery in facultative HC after ZO-2 transfection. We suspect that the effect was diluted because only a small percentage of cells were transfected (Figure 4e). 

Our results indicate that the lack of ZO-2 triggers a decrease in the content of constitutive and facultative HC that might favor the altered nuclear shape found in ZO-2 KD cells.

### 3.6. The Nuclei of ZO-2 KD MDCK Cells Are Lobulated and Present Indentations Due to the Instability of Microtubules

**Figure 5:**
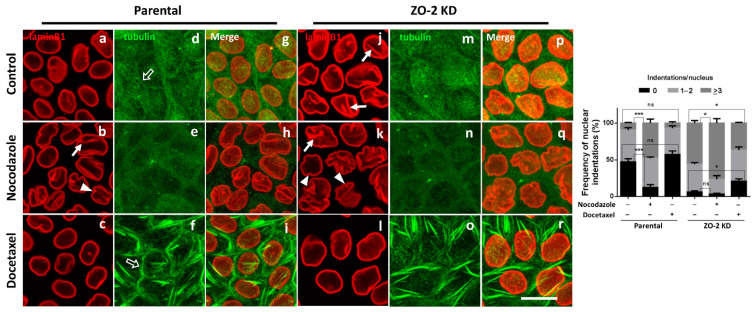
Stabilization of microtubules with docetaxel inhibits nuclear lobulation and indentation in ZO-2 KD MDCK cells. Parental and ZO-2 KD MDCK cells were incubated for 4 h with 10 μM nocodazole or with 10 μM docetaxel. Monolayers were fixed and processed for immunofluorescence with antibodies against lamin B1 and tubulin. Left panel, bar, 20 μm; full arrows, nuclear lobulations; full arrowheads, nuclear indentations; empty arrows, microtubules at cell borders. Right panel, quantitative analysis. Statistical analysis was done with One-way ANOVA followed by Tukey’s multiple comparisons test. Results shown as media ± standard deviation. ns, non-significant, * *p* < 0.05, *** *p* = 0.001.

Besides HC, nuclear lamina, and lamin B receptors, changes in nuclear shape can also result from an altered cytoskeleton. Therefore, we analyzed if the changes in nuclear shape observed in ZO-2 KD cells could be modulated by the microtubule cytoskeleton. In parental MDCK cells, treatment with 10 μM nocodazole that inhibits microtubule assembly [37] induces the appearance of nuclear lobulations and indentations (Figure 5a,b, left panel), whereas 10 μM of the taxane docetaxel that stabilizes microtubules [38] exerted no effect in nuclear shape (Figure 5a,c, left panel). Instead, in ZO-2 KD MDCK cells, treatment with docetaxel eliminated the lobulation and indentation of nuclei (Figure 5j,l, left panel), while nocodazole increased their lobulation and indented appearance (Figure 5j,k, left panel). Additionally, to prove the correct function of the drugs employed, we stained parental and ZO-2 KD MDCK cells with antibodies against tubulin. We observed that after nocodazole treatment, the planar apical network of non-centrosomal microtubules that laterally associate to TJs in parental monolayers [39] disappears (Figure 5d,e, left panel), whereas, after treatment with docetaxel, thick bundles of microtubules surround the cell borders (Figure 5 left panel, f), as has been previously reported [40]. In ZO-2 KD cells, we observed, as previously reported [11], that the microtubules are not laterally associated with the cell borders (Figure 5 left panel, m), even after treatment with docetaxel (Figure 5 left panel, o). The quantitative analysis of the indentations confirms that in parental cells, treatment with nocodazole diminishes the frequency of nuclei with zero indentations and augments those with three or more indentations, whereas in ZO-2 KD cells, docetaxel treatment increases the frequency of nuclei with zero indentations and reduces the percentage of nuclei with three or more indentations (Figure 5, right panel).

Our observations suggest that in ZO-2 KD cells, the altered nuclear shape is a consequence of microtubule instability.

### 3.7. ZO-2 Is Needed for the Establishment of the Microtubule Network between the Nucleus and the TJs That Maintains the Nuclear Shape

**Figure 6:**
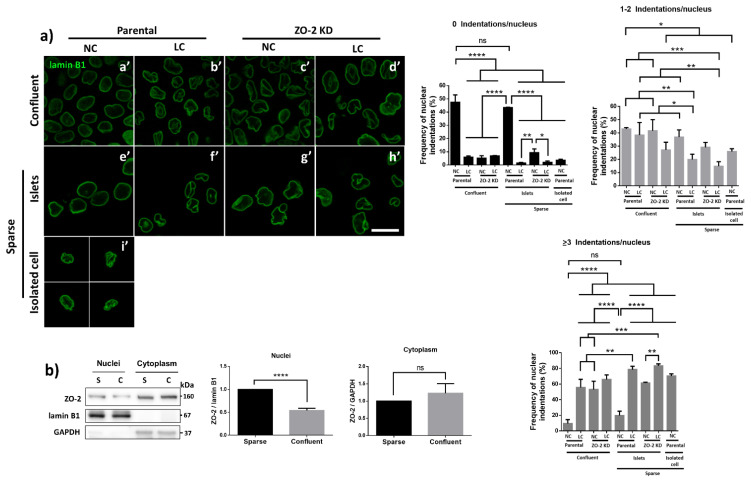
The nuclear shape is altered when the cells are cultured in LC, lack ZO-2 or are devoid of cell–cell contacts. (**a**) Immunofluorescence of lamin B1, in sparse or confluent cultures of parental and ZO-2 KD cells, cultured for 24 h in low calcium (LC, 1–5 μM Ca^2+^) or normal calcium (NC, 1.8 mM) media. Left panel, representative images; right panels, quantitative analysis of nuclear indentations. Statistical analysis was done with One-way ANOVA followed by Tukey’s multiple comparisons test. Results are shown as media ± standard deviation. ns, non-significant; * *p* < 0.05; ** *p* = 0.01; *** *p* = 0.001; **** *p* < 0.0001. (**b**) Western blot analysis of ZO-2 present in cytoplasmic and nuclear fractions derived from sparse (S) and confluent (C) cultures of parental MDCK cells. Lamin B1 and GAPDH were employed as markers and loading controls for nuclear and cytoplasmic fractions, respectively. Left, representative images; right quantitative analysis. Results from three independent experiments. Statistical analysis was done with Student’s *t*-test. Results are shown as media ± standard deviation. ns, non-significant; **** *p* < 0.0001.

Previously, we showed that the absence of ZO-2 diminishes the phosphorylation by AMPK (adenosine monophosphate-activated protein kinase) of cingulin, a TJ adaptor protein [11], critical for the association of microtubules to the TJ [39]. Hence, ZO-2 KD cells cultured in NC media (1.8 mM Ca^2+^) have sealed TJs, but lack the apical network of microtubules that associates to the cell borders [11]. These observations, together with our results showing that microtubule stabilization with docetaxel eliminated the nuclear lobulations and indentations in ZO-2 KD cells, prompted us to test if the change in nuclear shape could also be observed in cells that lack TJs due to their culture in low calcium (LC, 1–5 μM Ca^2+^) media [41,42], or because they are isolated due to low density plating. The immunofluorescence of lamin B1 reveals that both parental (Figure 6a(b’,f’)) and ZO-2 KD cells (Figure 6a(d’,h’)) cultured in the LC condition exhibit an altered nuclear shape in confluent and sparse cultures, similar to ZO-2 KD cells cultured in NC (Figure 6a(c’,g’)). A normal nuclear shape is present in confluent cultures and islets of parental cells in the sparse cultures maintained in NC (Figure 6a(a’,e’)). Instead, the form of the nucleus is altered in single parental cells cultured in NC that cannot establish TJs due to the lack of cell–cell contacts (Figure 6a(i’)). Hence, our observations suggest that the presence of a regular nuclear shape requires a network of microtubules connecting the cell borders to the nucleus, and for this purpose the presence of ZO-2 appears to be needed at both the TJ and the nucleus.

In epithelial cells in culture, the presence of ZO-2 at the nucleus diminishes as the culture becomes confluent and the protein departs from the nucleus to the cell borders [5,43]. Therefore, we next tested if confluent parental cells cultured in NC, maintain a certain amount of ZO-2 at the nucleus. The Western blot in Figure 6b reveals that a small amount of ZO-2 is maintained in the nucleus of confluent parental MDCK cells. As expected by previous results [5,43], this amount of ZO-2 is lower than that found in the nuclei of sparse cultures. These observations hence suggest that the presence of TJs at the cell borders, together with a small amount of nuclear ZO-2, are required to maintain the nuclear shape.

### 3.8. ZO-2 Associates to SUN-1 and Lamin B1, and Its Absence Alters the Expression of Nesprins-3 and -4 and Vimentin

**Figure 7:**
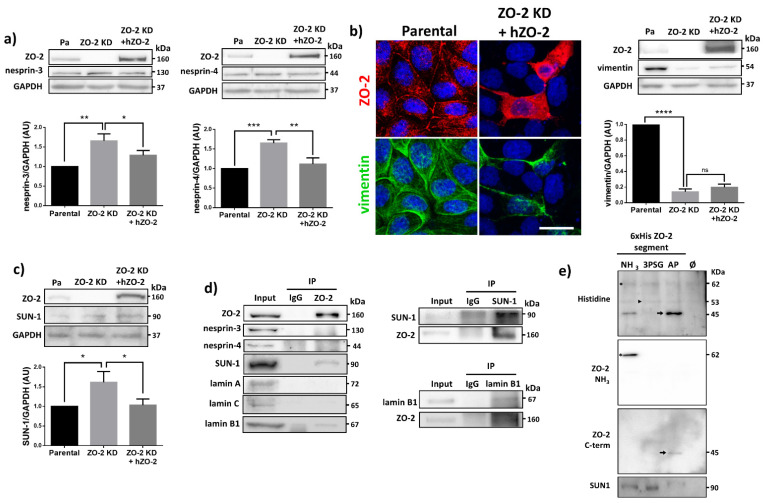
ZO-2 associates to SUN-1 and lamin B1, and its absence triggers an increased expression of SUN-1 and nesprins-3, and -4 and reduces vimentin. (**a**) Western blot detection of nesprins-3 and -4 in parental and ZO-2 KD cells transfected or not with hZO-2. Upper panels, representative images; lower panels, quantitative analysis obtained from three independent experiments. GAPDH is used as a loading control. Statistical analysis was done with One-way ANOVA followed by Tukey’s multiple comparisons test. Results shown as media ± standard deviation, * *p* < 0.05, ** *p* < 0.01, *** *p* < 0.001. (**b**) Left panel, representative immunofluorescence images showing ZO-2 and vimentin expression in parental and ZO-2 KD cells transfected or not with hZO-2. Bar, 20 μm. Right panel, representative Western blot of three independent experiments and corresponding quantitative analysis. GAPDH is used as a loading control. Statistical analysis was done with One-way ANOVA followed by Tukey’s multiple comparisons test. Results are shown as media ± standard deviation, **** *p* < 0.0001; ns, non-significant. (**c**) Western blot detection of SUN-1 in parental and ZO-2 KD cells transfected or not with hZO-2. Upper panel, representative image; lower panel, quantitative analysis obtained from three independent experiments. GAPDH is used as a loading control. Statistical analysis was done with One-way ANOVA followed by Tukey’s multiple comparisons test. Results are shown as media ± standard deviation, * *p* < 0.05. (**d**) Left column, immunoprecipitation of ZO-2 from sparse MDCK cells followed by Western blot detection of ZO-2, nesprins-3 and -4, SUN-1, and lamins A, C and B1. Right panels, immunoprecipitation of SUN-1 and lamin B1 from sparse MDCK cells followed by Western blot detection of ZO-2, SUN-1, and lamin B1. Representative image from three independent experiments. (**e**) 6xHis-tagged amino (NH3), 3PSG and AP segments of cZO-2 and the empty vector (Φ, negative control) were purified with Ni affinity columns from extracts of HEK293T cells, run in SDS-PAGE and blotted with antibodies against the histidine tag, the amino and C-terminal portion of ZO-2, or pulled-down SUN-1 protein. Upper panel, * band of 61 kDa of amino ZO-2 segment; full arrowhead, band of 53 kDa of 3PSG ZO-2 segment; full arrow, band of 45 kDa of AP ZO-2 segment. Second panel from top, * band of 61 kDa of amino ZO-2 segment. Third panel from top, full arrow, band of 45 kDa of AP ZO-2 segment. Lower panel, anti SUN-1 antibody gives a positive signal in pull-downs of amino and 3PSG segments of cZO-2.

We then explored if the lack of ZO-2 altered the expression of proteins of the LINC complex that provide nuclear stability by crosslinking the nuclear lamins to the cytoskeleton. For this purpose, we analyzed the expression of nesprins-3 and -4, finding that they augment in ZO-2 KD cells and return to control levels upon transfection with an hZO-2 construct resistant to the anti-ZO-2 shRNAs (Figure 7a). Since nesprin-3 connects via plectin to intermediate filaments in the cytoplasm [44], we next analyzed vimentin expression, a type II intermediate filament. We observed that vimentin expression almost disappears in ZO-2 KD cells by immunofluorescence and is re-established in these cells upon transfection with hZO-2 (Figure 7b). By Western blot, we confirmed in ZO-2 KD cells the decreased vimentin expression but could not detect the increase in vimentin triggered by hZO-2 transfection due to the low number of transfected cells.

Additionally, we explored if the expression SUN-1 that connects lamin A to nesprins was altered in the ZO-2 KD cells, finding an increased expression of this protein that returned to control values after ZO-2 transfection (Figure 7c). We then asked if ZO-2 associates with the LINC complex, and for this purpose, we immunoprecipitated ZO-2 from sparse MDCK cells and detected by Western blot the presence of SUN-1, nesprins-3 and -4, and lamins A, C, and B1. Figure 7d reveals that while nesprins-3 and -4 and lamins A and C do not co-immunoprecipitate with ZO-2, SUN-1 does co-immunoprecipitate with ZO-2 and confirmed the previous observation of the co-immunoprecipitation of lamin B1 with ZO-2 [45]. To further confirm these results, we immunoprecipitated SUN-1 or lamin B1 and, by Western blot, detected the presence of ZO-2 in the immunoprecipitate.

To reinforce the observation of the interaction between SUN-1 and ZO-2, we made a pull-down assay in the human kidney cell line HEK293T. For this purpose, the cells were transfected with the amino (coding PDZ domains 1, 2, and 3), 3PSG (coding PDZ-3, SH3, and GuK domains) or AP (coding the acidic and proline-rich regions) constructs of cZO-2, introduced in the pcDNA4/HisMax vector. The corresponding proteins were purified from extracts of HEK293T cell using Ni affinity columns, run in SDS-PAGE, and blotted with antibodies against the histidine tag. The upper panel of Figure 7e reveals bands of 62, 53 and 45 kDa that respectively correspond to the amino, 3PSG and AP segments of cZO-2. The identity of the amino pulled-down segment was further confirmed in a blot with an anti-ZO-2 antibody that recognizes the amino section of the protein (Figure 7e, second panel from top) and another antibody against the C-terminal portion of ZO-2 (Figure 7e, third panel from top). The identity of the 3PSG segment of ZO-2 could not be confirmed with a specific antibody since there is no commercial antibody for it. We then detected, with an antibody against SUN-1, the presence of this protein in the pull-down of the amino and 3PSG segments of ZO-2. Since both of these segments have in common the third PDZ domain of ZO-2, it is possible that SUN-1 associates with ZO-2 PDZ-3 domain (Figure 7e, lower panel). Altogether, these results indicate that at the nucleus, ZO-2 acts as a scaffold that associates with lamin B1 and SUN-1.

**Figure 8:**
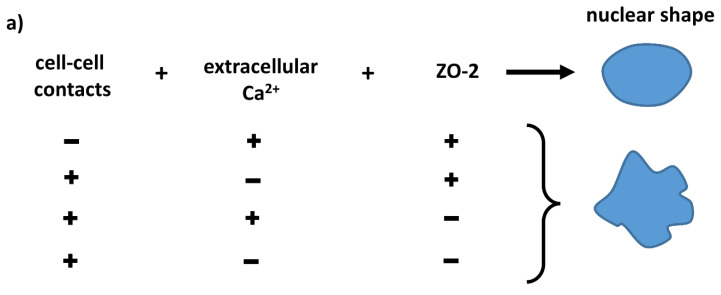

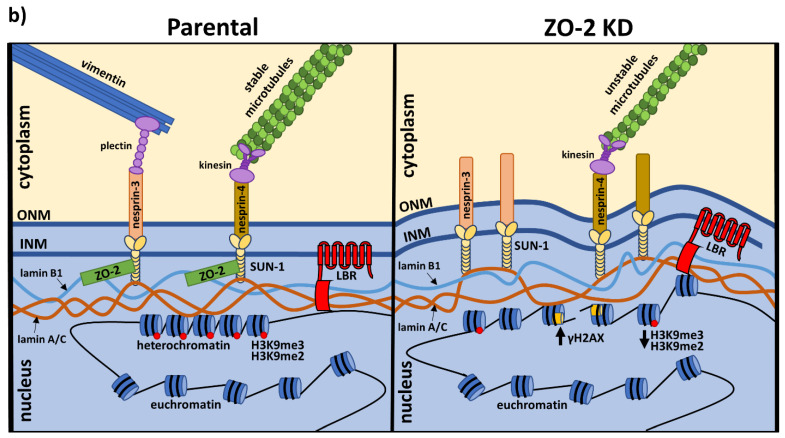
Schematic representation of main observations. (**a**) Cell-cell contacts, extracellular calcium, and ZO-2 are required by epithelial cells to maintain the normal nuclear shape. (**b**) ZO-2 association to proteins at the inner nuclear membrane, which forms part of the LINC complex that links the nucleoskeleton to the cytoskeleton. ZO-2 at the nucleus associates with lamin B1 and SUN-1. The latter is an inner nuclear membrane protein that associates with nesprins-3 and -4, which, through plectin and kinesin, respectively, bind to vimentin and microtubules. In the absence of ZO-2, the nuclei become lobulated with multiple indentations; constitutive and facultative markers of HC H3K9me3 and H3K9me2 respectively diminish, and DNA DSBs, identified with ϒ-H2AX, are more abundant than in parental cells. These changes are accompanied by an over-expression of SUN-1 and nesprins-3 and -4; a diminished expression of vimentin; and instability of microtubules. These observations suggest that the contact of ZO-2 with proteins in the inner nuclear membrane is crucial for the maintenance of the nuclear shape. ONM, outer nuclear membrane; INM, inner nuclear membrane; LBR, lamin B receptor.

In summary, we have found that ZO-2 and cell–cell contacts mediated by extracellular calcium are required for normal nuclear shape (Figure 8a). The lack of ZO-2 alters nuclear morphology and increases DNA DSBs, but does not induce senescence. The change in nuclear shape is not accompanied by an altered expression of lamins A/C and B1, or lamin B receptor, and instead involves a decrease in constitutive and facultative heterochromatin. Moreover, the results reveal that ZO-2 associates with the nucleoskeleton of lamin B1 and the INM protein SUN-1 (Figure 8b).

## 4. Discussion

Here, we have observed that, in the absence of ZO-2, epithelial MDCK cells display lobulated nuclei with multiple indentations. This change in nuclear shape and the previously described increase in cell size in ZO-2 KD cells [10] prompted us to explore if these cells are senescent. We examined β-galactosidase activity, lamin B1 immunofluorescence, and the amount of p21 detected in UV-irradiated cells to reveal that ZO-2 KD cells are even less committed towards UV-induced senescence than parental cells. This observation agrees with a previous finding showing that ZO-2 KD cells grow at a standard rate [10]. The present results also reveal in ZO-2 KD cells an unaltered level of lamin B1, which is regularly depleted in senescent cells [46,47]. In addition, we found a minimal vimentin expression, which in senescent cells is much higher than in actively growing cultures [48]. Senescence is a critical feature of mammalian cells for preventing tumor formation (for reviews, see [49,50,51]). Hence, the resistance to senescence displayed by epithelial cells lacking ZO-2 might further support the emerging idea of ZO-2 as a tumor suppressor protein (for review see [52]).

Besides ZO-2 KD cells, we also found an irregular nuclear shape in cells that lack TJs because they are cultured in LC medium or lack neighbors due to extreme low density plating. These observations highlight the importance of the interaction of the nucleoskeleton with the cytoskeleton that reaches the TJ to maintain the nuclear shape. Moreover, these results indicate that the presence of ZO-2 at the nucleus and TJs is required to preserve the nuclear shape.

Lack of ZO-2 triggers an increase in DNA DSBs, evaluated by an augmented expression of ϒ-H2AX nuclear foci. We suspect that they could arise due to deformed nuclei where the lack of vimentin and instability of microtubules can no longer provide proper mechanical resistance to the nucleus. In agreement, an increase in ϒ-H2AX nuclear foci has been observed in vimentin-null mouse embryonic fibroblasts compared to vimentin expressing cells, after migration through small pores [53].

Changes in nuclear cell shape from ovoid to lobulated are observed during the differentiation of peripheral blood granulocytes [54]. In human leukemic cells treated with retinoic acid (RA), this process is accompanied by a decrease in the amount of lamins A/C and B1, vimentin, and an increase in the lamin B receptor thought to augment the flexibility of the nuclear envelope [33]. However, in ZO-2 KD MDCK cells, we did not observe a difference with parental cells in the expression of lamins A/C and B or lamin B receptors, but we did find a diminished expression of vimentin. Likewise, in mouse embryonic fibroblasts that do not express vimentin, migration through small pores elongates the nuclear shape but does not change the expression levels of lamins A/C and B1/2 [53].

In ZO-2 KD cells, the dramatic decrease in vimentin expression could play a significant role in the change of nuclear shape, as this intermediate filament enhances perinuclear cell stiffness [55]. Vimentin forms a cage around the nucleus that constricts along with the nucleus during motility through confined spaces and provides mechanical resistance against strains that could damage cell integrity. Hence, in mammalian cells, vimentin, lamins A/C and SUN are needed to restore the nuclear shape and position after a nuclear force displacement [56].

Here, we also analyzed chromatin alterations that could disrupt nuclear shape. We focused on chromatin histone modifications, which affect nuclear morphology independent of lamins [35]. In this respect, histone demethylase inhibitors that increase HC augment nuclear rigidity, which results in a reduced nuclear blebbing [35]. Since an increased HC stiffens the nuclei of epithelial cells [34], we analyzed in the nuclei of parental and ZO-2 KD cells the expression of the constitutive and facultative heterochromatin markers H3K9me3 and H3K9me2, respectively. The reduced expression of these markers in ZO-2 KD cells suggests that nuclear shape disruption could result from less HC that generates softer nuclei.

We observed that upon treatment with docetaxel that increases microtubules polymerization [38], the nuclei of ZO-2 KD MDCK cells acquire a shape similar to that present in parental cells. These results, hence, suggest that the lack of ZO-2 produces instability of microtubules. In accordance, in ZO-2 KD cells, we found an altered expression of nesprin-4, which through kinesin binds to microtubules (for review, see [18]). In this regard, we had previously observed that ZO-2 KD MDCK cells lose the microtubules that in parental cells are aligned laterally to TJs, forming a planar apical structure [11]. We also formerly found that the presence of ZO-2 facilitates the interaction of the TJ adaptor protein cingulin with microtubules at the apical junctional region [11]. Moreover, our present results agree with previous observations showing that microtubules influence the nuclear shape. Thus, in leukemic HL-60 cells, the addition of retinoic acid (RA) induces the development of lobulated nuclei that mimic nuclear differentiation during granulopoiesis. However, in this model, in contrast to our results, nocodazole prevents RA induction of lobulation, whereas taxol induces lobulation even in the absence of RA [57]. The relation of the nucleus and microtubules is also highlighted by the observation that microtubules envelop the entire nucleus, and disruption or excess formation of stable microtubules block nuclear movement during neuronal migration in the developing brain [58].

The increased expression of nesprins-3 and -4, together with the negligible amount of vimentin and the instability of microtubules observed in cells lacking ZO-2, strongly suggests an altered link between lamin B1 and the cytoskeleton of intermediate filaments when ZO-2 is absent. This agrees with our previous observation showing the interaction in a pull-down assay of ZO-2 fusion proteins 3PSG and AP with lamin B1 [45].

It is noteworthy that ZO-2 co-immunoprecipitates with lamin B1 but not with lamins A and C. This might rely on the fact that lamin B1, in contrast to lamin A/C forms an outer ring within the nuclear lamina localizing closest to the INM [15]. The lost interaction between ZO-2 and lamin B1 in ZO-2 KD cells may also contribute to the altered nuclear shape, since lamin B1 stabilizes nuclear shape by restraining outward protrusions of the lamin A/C network [15].

The observations that the expression of SUN-1 augments in ZO-2 KD cells, that SUN-1 co-immunoprecipitates with ZO-2 and that it is present in a ZO-2 pull-down assay, suggests a role for ZO-2 as a scaffold for the LINC complex in the INM of the nuclear envelope. However, more evidence will be needed in the future to explain the role of ZO-2 on the LINC complex and on nuclear shape.

Our results agree with previous observations showing by Western blot the presence of ZO-2 in a nuclear matrix preparation devoid of soluble nuclear proteins [32]. Moreover, they coincide with previous immunofluorescences showing ZO-2 at the nucleus of fixed cells subjected to a treatment that sequentially released soluble proteins with Triton X-100, the salt-labile cytoskeleton with ammonium sulfate, and the chromatin-associated proteins with DNAse and RNAse [45].

Nesprin-3 is required in Sertoli cells of the testis, to localize vimentin at the nuclear perimeter [59]. Here, we have observed that in MDCK ZO-2 KD cells an almost absent expression of vimentin is accompanied by an increased expression of nesprin-3, further supporting their functional link. In nesprin-3 knock-out (KO) mice no obvious phenotype is present [59]. Instead, KO mice of nesprin-4 or SUN-1 developed progressive hearing loss, as the outer hair cells of the inner ear degenerated and failed to maintain their nuclei in a basal position, concluding that the LINC complex is essential for hearing [60]. Interestingly, in humans, autosomal dominant non-syndromic hearing loss is associated with mutations in the *TJP2* gene that codes for ZO-2 [61,62]. In mice, a mutation in the catalytically inactive deubiquitinating enzyme USP53, which interacts with ZO-2 in cochlear hair cells, also induces progressive hearing loss [63]. These observations suggest that hearing loss related to mutations in *TJP2* and associated proteins might also be related to alterations in the LINC complex.

In summary, we have found that cell–cell contacts mediated by extracellular calcium, and ZO-2 are required by epithelial cells to maintain the normal nuclear shape, and that ZO-2 at the nucleus associates to the nucleoskeleton protein lamin B1 and to SUN-1, an INM protein of the LINC complex.

## Supplementary Materials

The following are available online at https://www.mdpi.com/article/10.3390/cells10102568/s1, Figure S1: Nuclear lobulations and indentations are present in the three clones of ZO-2 KD MDCK cells; Figure S2: Transfection efficiency of MDCK cells; Figure S3: The monolayers of parental and ZO-2 KD MDCK cells do not exhibit apoptotic cells before or after UV-radiation.
