# Supplementary material for: Zonula occludens 2 and Cell-Cell Contacts Are Required for Normal Nuclear Shape in Epithelia
Source: Cells. 2021 Sep 28;10(10):2568. doi: 10.3390/cells10102568 (PMC8534263; doi:10.3390/cells10102568)
Supplement: Supplementary file 1 [file cells-10-02568-s001.zip › cells-1368728-supplementary.pdf]

Supplementary Material

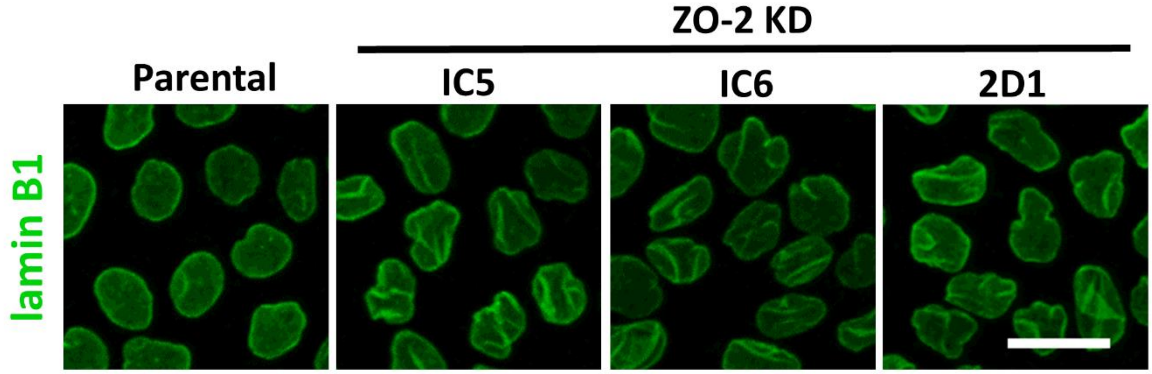

Figure S1. Nuclear lobulations and indentations are present in the three clones of ZO-2 KD MDCK cells. Parental MDCK cells and three clones of ZO-2 KD cells were treated with an antibody against lamin B1. Bar, 20 μm.

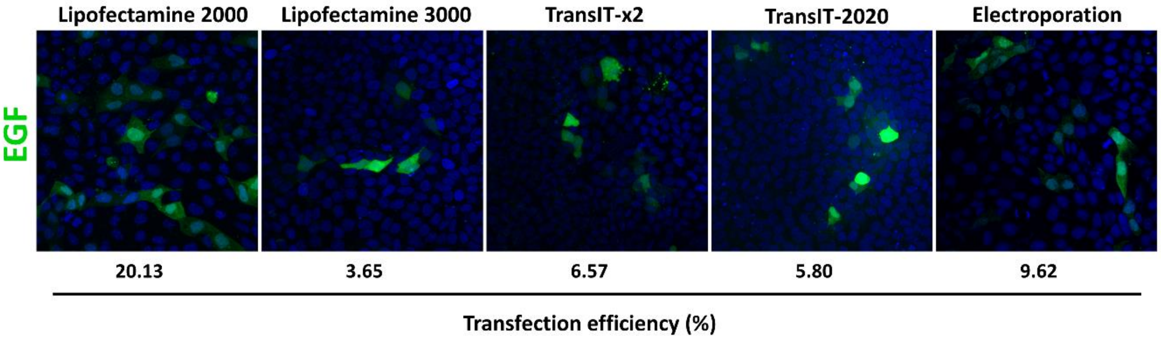

Figure S2. Transfection efficiency of MDCK cells. MDCK ZO-2 KD cells were transfected with an enhanced green fluorescent (EGF) construct employing electroporation or different transfection reagents. The number of transfected cells observed by immunofluorescence in three optical fields per condition was used to calculate transfection efficiency.

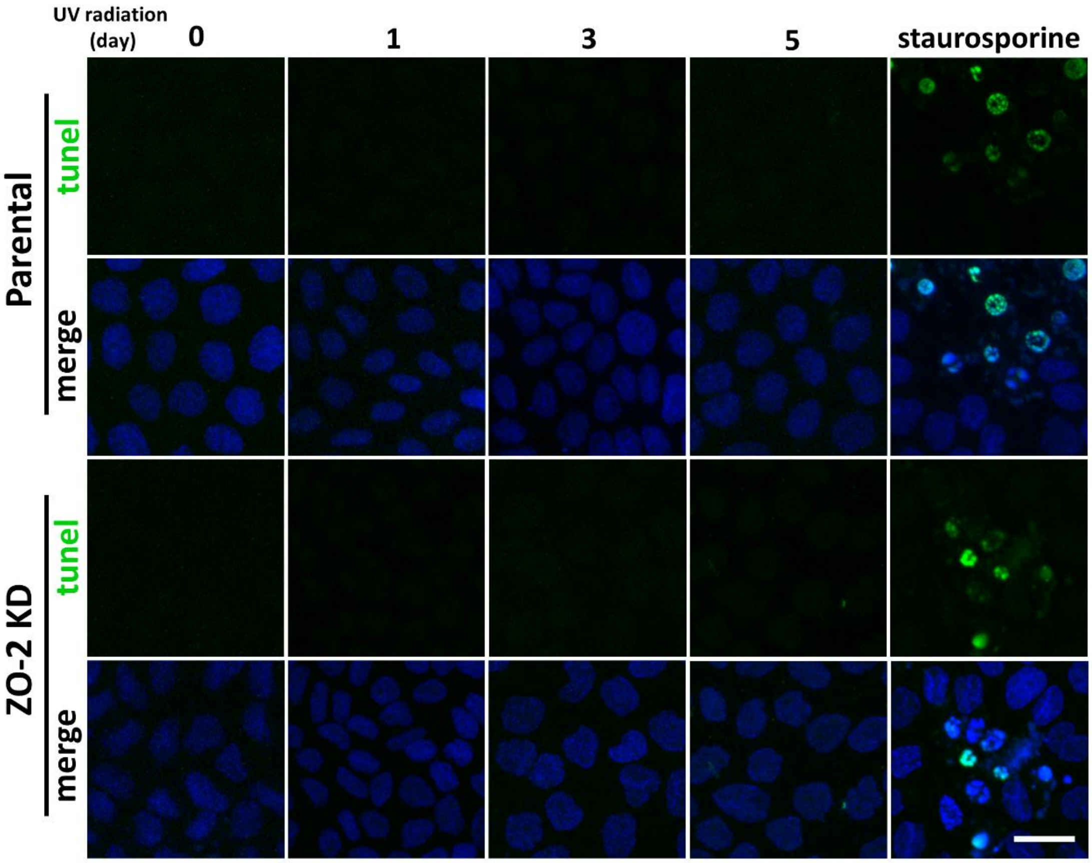

Figure S3. The monolayers of parental and ZO-2 KD MDCK cells do not exhibit apoptotic cells before or after UV-radiation. TUNEL assay for apoptosis done in ZO-2 KD and parental MDCK monolayers subjected to UV radiation. Treatment with 2 μM staurosporine for 24 h was used as positive control.

.
